# Does in Ovo Injection of Two Chicken Strains with Royal Jelly Impact Hatchability, Post-Hatch Growth Performance and Haematological and Immunological Parameters in Hatched Chicks?

**DOI:** 10.3390/ani9080486

**Published:** 2019-07-25

**Authors:** Ayman E. Taha, Osama A. AbdAllah, Khalil M. Attia, Ragaa E. Abd El-Karim, Mohamed E. Abd El-Hack, Mohamed A. El-Edel, Islam M. Saadeldin, Elsayed O. S. Hussein, Ayman A. Swelum

**Affiliations:** 1 Department of Animal Husbandry and Animal Wealth Development, Faculty of Veterinary Medicine, Alexandria University, Behira, Rasheed, Edfina 22758, Egypt; 2 Animal Production Research Institute, Ministry of Agriculture, Dokki, Cairo 12618, Egypt; 3 Department of Poultry, Faculty of Agriculture, Zagazig University, Zagazig 44511, Egypt; 4 Department of Animal Husbandry and Animal Wealth Development, Faculty of Veterinary Medicine, Damanhour University, Damanhour 22511, Egypt; 5 Department of Animal Production, College of Food and Agriculture Sciences, King Saud University, P.O. Box 2460, Riyadh 11451, Saudi Arabia; 6 Department of Physiology, Faculty of Veterinary Medicine, Zagazig University, Zagazig 44511, Egypt; 7 Department of Theriogenology, Faculty of Veterinary Medicine, Zagazig University, Zagazig 44511, Egypt

**Keywords:** strain, in ovo injection, royal jelly, chicken, growth, hatchability, blood

## Abstract

**Simple Summary:**

The present investigation examined improvements in egg hatchability and the growth performance of hatched chicks of two strains upon injection with increasing concentrations of royal jelly (RJ). The results showed positive effects of RJ injection on all parameters. Limited impacts of the different chicken strains were observed on the tested parameters. The study revealed that varying the chicken strain could alter the response to the in ovo injection with RJ.

**Abstract:**

The hypothesis of the present work was that the effects of in ovo injection may differ in different chicken strains. The influence of in ovo royal jelly (RJ) injection on hatching, growth and blood parameters in two chicken strains (Dokki-4 and El-Salam as example for different strains) was evaluated. A total of 1080 eggs were used. On the seventh day of incubation, the eggs were randomly allocated into six experimental groups in a 2 × 3 arrangement that included the two chicken strains and three concentrations of RJ (0, 0.25 and 0.5 mL RJ/egg). Injection with 0.5 mL RJ/egg improved hatchability compared to the other treatments. The El-Salam strain exhibited significantly higher body weight and body weight gain than the Dokki-4 strain. Injection with 0.5 mL RJ/egg significantly (*p* < 0.05) improved chicken body weight and daily weight gain compared to the control treatment. RJ injection decreased blood lipid profile parameters and the numbers of monocytes and eosinophils and increased total protein, globulin, haemoglobin (Hb) and lymphocyte levels compared to the control treatment. The Dokki-4 strain showed significantly higher antibody titres against avian influenza virus (AIV) (*p* < 0.05) and sheep red blood cells (SRBCs) (*p* < 0.0001) than the El-Salam strain and RJ injection enhanced antibody titres against AIV, Newcastle disease virus (NDV) and SRBCs. Therefore, the Dokki-4 strain was superior to the El-Salam strain for the tested parameters and injection with 0.5 mL RJ/egg produced the best hatching parameters, growth performance and health-related traits. RJ in ovo injection was much more effective in the Dokki-4 strain than in the El-Salam strain, which supported the hypothesis of the study that varying the chicken strain could alter the response to the in ovo injection with RJ.

## 1. Introduction

Embryonic growth in poultry can be manipulated through in *ovo* administration of nutrients and natural bioactive compounds [1,2,3,4,5,6,7]. In ovo injection of such substances influences the pre- and post-hatching physiological status of broiler embryos, leading to improved hatchability, superior nutritional status of hatchlings, greater vigour and higher post-hatch growth [8,9].

Royal jelly (RJ), a honeybee secretion fed to larvae and queen bees, is a rich dietary supplement for humans. RJ in fresh form consists of water (60–70%), protein (9–18%), carbohydrate (7–18%), fat (3–8%), mineral salts (calcium 1.5%), 10-hydroxy-2-decenoic acid (1.4%), fructose (3–13%), glucose (4–8%), sucrose (0.5–2.0%) and Ash (0.8–3.0%). While the lyophilised form contains <5% water, 27–41% protein, 22–31% carbohydrate and 15–30% fat [10,11].

RJ is the richest known natural source of vitamin B5 [12]. The other components of RJ include gamma globulins, mostly immunoglobulins, which powerfully strengthen the immune system; 10-hydroxy-∆2-decanoic acid, which is a powerful antibacterial and anti-fungal agent [13] that keeps RJ sterile; gelatine, the precursor of collagen in skin, tendon, ligaments; and up to 1 mg/g acetylcholine, of which RJ is the richest natural source and which important in nerve transmission and the production and release of glandular secretions [14].

RJ has been reported to have several pharmacological properties, such as antioxidant [15], hypocholesterolaemic [16], anti-inflammatory [17], anti-malignant [18], antibacterial [19] and anti-ageing [20] properties, in animals. Additionally, RJ in ovo injection has been concluded to improve chick body weight [21], internal organ weight and luteinizing hormone (LH) and follicle-stimulating hormone (FSH) secretion without adverse effects on hatchability [22]. Furthermore, in ovo injection of RJ has been found to promote feed intake in broiler chicks with no effect on immunity against Newcastle disease virus (NDV) [23], egg quality parameters or erythrocyte counts [24].

Different strains of chickens differ in their productivity, reproductive performance and immune responses [25,26,27,28]. Furthermore, strain differences affect feed intake, feed conversion ratios (FCRs) [29] and carcass traits [30,31].

Several studies have reported the effects of in ovo injection on the pre- and post-hatching performance of chickens; however, interactions between strain and in ovo injection have not been investigated. Therefore, the present study aimed to analyse the effects of in ovo injection of different levels of RJ, strain differences and interactions between strain and RJ treatment levels on hatching, growth rates, blood chemistry, haematology and immunological parameters.

## 2. Materials and Methods

This study was carried out at the Poultry Farm, Faculty of Agriculture, Kafr El-Sheikh University, Egypt. All procedures and experiments were performed in accordance with the ethical guidelines of the Committee of Local Experimental Animal Care and were approved by the Faculty of Agriculture, Kafr El-Sheikh University, Egypt (KFS2018-0078). All efforts were made to minimize the suffering of the animals.

A total of 1080 incubating eggs produced by two local chicken strains (Dokki-4 and El-Salam) were used. The Dokki-4 strain was developed by El-Itriby and Sayed [32] from a cross between a Fayoumi cock and Barred Plymouth Rock females (used as a dual purpose, meat and egg production) and the El-Salam strain was developed by Abd El-Gawad et al. [33] from crosses between Nichol sires and Mamourah dams (used as a dual purpose, meat and egg production).

The eggs were collected from the Experimental Research Station, Sakha, of the Animal Production Research Institute, Agricultural Research Centre, Ministry of Agriculture, Egypt. The eggs were stored at 15 ± 1 °C with 70–75% relative humidity for 3 days. The eggs were immediately cleaned with a dry, clean cloth; then, the surface of each egg was sprayed with a disinfectant solution and the shell was wiped dry with clean paper. The eggs were incubated at 37.6 ± 1 °C with a relative humidity of 57 ± 2%. All eggs were set large end up in an automatic turner. The incubation equipment included an incubator (for the first 18 days of incubation) and a hatcher (for the remaining 3 days until hatching). The eggs from each experimental group were set in separate and marked containers in both the incubator and the hatcher.

On day 7 of incubation, the eggs were randomly divided into six groups in a 2 × 3 factorial design that included the two chicken strains (Dokki-4 and El-Salam) and three levels of in ovo injections (0, 0.25 and 0.5 mL/egg). Each group had six replicates of 30 eggs each. The RJ solution was prepared by dissolving 1 g of pure RJ (YS Organic Bee Farms “2774 N 4351st Rd, Sheridan, IL 60551, USA,” item #69313, 3x freeze-dried RJ; equivalent to 1500 mg of fresh RJ) in 2 mL of normal saline solution in a water bath at 37 °C for 15 min.

A hole was made in the broad end of each egg using an automatic needle (syringe); 0.5 mL of RJ solution was then warmed to 30 °C and injected through the hole using a 23–gauge needle. The injection site was disinfected with 70% ethanol prior to injection. The pinhole was sealed with sterile paraffin wax immediately after injection and the eggs were returned to the incubator and set large end up to complete the incubation process. At the end of 18 days of incubation, the eggs were transferred to hatching trays at 37.2 °C under 70% humidity for the following 3 days or until hatching. After 21 day of incubation, the live hatched chicks were graded and counted, while the un-hatched eggs were broken to estimate the percentage of fertility. Hatchability was calculated as a percentage of fertile eggs using the following equations:

(1) Fertility (%)=(fertile eggs/total eggs)×100

(2) Hatchability of the fertile eggs (%)=(hatched chick/fertile eggs)×100

Twenty hatched chicks for each replicate were sexed, wing-banded and reared until 12 weeks of age and were supplied with standard feed for local chicken strains. The basal diet contained 19% crude protein (CP), 2834 kcal/kg metabolizable energy (ME), 3.019% ether extract, 3.906% crude fibre, 1.018% calcium, 0.348% available phosphorus, 0.857% lysine, 0.360% methionine and 0.699% methionine and cystine. The birds were vaccinated against most epidemic diseases in Egypt; they were vaccinated with Hitchner B1 (HB1) and Gumboro vaccines at 7 and 10 day of age, respectively, via eye drops and killed NDV, Reo, Gumboro and infectious bronchitis (IB) vaccines were injected intramuscularly at 13 day of age. Killed avian influenza virus (AIV; H5N2) vaccines were injected intramuscularly at 15 day of age, while Gumboro and Lasota vaccines were given at 22, 32 and 42 day of age via eye drops. Later, booster doses of Lasota vaccines were given at 50 day of age and then biweekly via eye drops. Feed and drinking water were offered ad libitum. All birds were reared under the same environmental, managerial and hygienic conditions. Body weight was recorded to the nearest 0.1 g. Feed intake was recorded at 0, 4, 8 and 12 weeks of age and the FCR was then calculated.

At 7 weeks of age, the birds received a single intramuscular injection of 0.1 mL of a 0.25% sheep red blood cell (SRBC) suspension. After 5 days, ten blood samples (1 mL each) from each group were collected from the wing vein with a sterile syringe and 0.5 mL of each sample was transferred into a heparinized tube. Plasma antibodies were measured by the microtitre haemagglutination method [34]. The titres are expressed as the log2 of the reciprocal of the highest dilution in which there was haemagglutination. The remaining 0.5 mL of each blood sample was allowed to coagulate in sterile tubes, after which the serum was collected to assess the antibody titre against NDV.

At 12 weeks of age, ten birds (5 males and 5 females) were selected randomly from each treatment, weighed and then sacrificed by decapitation. The carcasses and giblets (gizzard, heart and liver) were individually weighed to the nearest 0.1 g. The studied carcass traits were recorded as the percentage of the live body weight. Ten blood samples were collected in heparinized tubes to determine the complete blood count (CBC) and white blood cell (WBC) differentiation. After overnight clotting at 4 °C, the samples were centrifuged for 20 min at 4000× *g*.

The separated serum was transferred to a private laboratory for analysis of biochemical parameters. The total lipids (mg/dL), cholesterol (mg/dL), triglycerides (TGs; mg/dL), high-density lipoprotein (HDL, mg/dL), low-density lipoprotein (LDL, mg/dL), total protein (g/dL) and albumen (g/dL) levels were determined spectrophotometrically according to the methods of Akiba et al. [35], using commercial diagnostic kits from Biodiagnostic Company, Giza, Egypt. Additionally, the antibody titres against AIV and NDV were estimated.

The data were subjected to ANOVA with the generalized linear model (GLM) procedure of SAS software, USA [36] according to the following model:

(3) $$X_{rtk}=µ+O_{r}+B_{f}+I_{r\times t}+E_{rtk}$$

where *X_rtk_* = the value of any observation, µ = the population mean *O_r_* = the in ovo injection effect (of 0, 0.25 or 0.5 mL RJ/egg), *B_f_* = the strain effect (El-Salam or Dokki-4), *I*_*r* × *t*_ = the interaction between the treatment and the chicken strain and *E_rtk_* = random error.

## 3. Results

### 3.1. Hatching Parameters

As shown in Table 1, the Dokki-4 strain presented significantly higher fertility than the El-Salam strain (*p* < 0.05). No differences were recorded in the hatchability of the total eggs and fertile eggs (*p* < 0.05) between the two studied strains. Regarding the effect of in ovo RJ injection, the 0.5 mL RJ/egg dose significantly increased the hatchability of the total incubated eggs (*p* < 0.0001) compared to both the 0.25 mL RJ/egg dose and the control dose (0 mL RJ/egg) and improved the hatchability percentages of fertile eggs (*p* < 0.05) compared to the control dose.

### 3.2. Growth Performance and Carcass Parameters

The results of chicken performance according to strain and in ovo injection level, as well as their interactions, are presented in Table 2. Daily weight gain was not significantly affected (*p* ≥ 0.05) by strain. The results of the interaction between strain and in ovo inoculation were not significant (*p* ≥ 0.05).

Feed consumption and conversion ratios did not differ significantly between the El-Salam and Dokki-4 strains, except for feed consumption at 0–12 weeks of age (*p* < 0.0001), which were significantly improved in the El-Salam strain. No variation in dressing percentages was observed between strains (*p* ≥ 0.05) but Dokki-4 had higher (*p* < 0.01) giblet percentages than the El-Salam Strain. For the in ovo RJ injection effects, chickens from eggs inoculated with 0.5 mL RJ consumed more feed (*p* < 0.05) compared to injection with 0.25 mL RJ or the control at 0–12 weeks of age; chickens from eggs inoculated with 0.5 mL RJ also displayed higher (*p* < 0.05) dressing percentages compared to the control group.

### 3.3. Serum Lipid and Protein Profiles

Strain clearly had no effect on serum lipid and protein profiles (Table 3). In contrast, in ovo injections with 0.5 mL RJ/egg decreased serum total lipids and increased (*p* < 0.001) globulin and total protein levels (*p* < 0.05) compared to the other injection. Both levels of RJ injection (0.25 and 0.5 mL RJ/egg) resulted in significantly reduced serum levels of cholesterol, TGs, HDL and LDL. No significant interactions between the strain and treatment effects were recorded for the serum lipid and protein profiles.

### 3.4. Complete Blood Count (CBC)

The effects of strain, RJ in ovo injection level and their interaction on CBCs are listed in Table 4. Strain had no significant effect (*p* > 0.05) on red blood cell (RBC) count; packed cell volume (PCV); haemoglobin (Hb); WBC count; heterophil and lymphocyte numbers; the heterophil/lymphocyte (HL) ratio; or monocyte, eosinophil and basophil numbers. With regard to the in ovo RJ injection levels, both 0.25 and 0.5 mL RJ/egg resulted in increased Hb levels and lymphocyte counts compared to 0 mL RJ/egg, whereas significant reductions were observed in the numbers of monocytes and eosinophils. No significant interactions were recorded between the strain and treatment effects for any of the blood parameters analysed.

### 3.5. Immunological Parameters

As illustrated in Table 5, the Dokki-4 strain had significantly higher antibody titres against the AIV vaccine (*p* < 0.05) and SRBCs (*p* < 0.0001) than the El-Salam strain but no significant differences (*p* > 0.05) were recorded between the two strains for NDV titres. Regarding the impact of in ovo RJ injection on the immunity of the chickens, both levels of RJ (0.25 and 0.5 mL RJ/egg) increased (*p* < 0.0001) the antibody titre against the AIV vaccine, while 0.5 mL RJ/egg increased (*p* < 0.0001) the antibody titres against NDV and SRBCs compared to the other treatments. Regarding the interaction between the strain and in ovo RJ injection effects, no significant differences in antibody titres against AIV and NDV were recorded between the different groups; however, in ovo injection with 0.25 or 0.5 mL RJ/egg increased immunity (*p* < 0.0001) against SRBCs in the Dokki-4 strain compared to the El-Salam strain.

## 4. Discussion

The data listed in Table 1 shows that the in ovo RJ injection (0.5 mL RJ/egg) improved the hatchability percentages of fertile eggs (*p* < 0.05) compared to the other groups. The improvement in hatchability may be due to the enriched nutritive values of RJ, which contain vitamins and essential amino acid that enhance chick embryonic growth and hatchability. However, our results disagree with those obtained by Moghaddam et al. [21] who reported that in ovo RJ injection significantly decreased hatchability (46.7%) compared to saline injection (68.3%). Moreover, Moghaddam et al. [22] found significantly lower hatchability percentages with RJ compared to saline phosphate antibiotic injection. Conclusively, RJ in ovo injections (0.5 mL RJ/egg) improved hatchability percentage of chicken eggs.

Our results showed that in ovo RJ injection with 0.5 mL RJ/egg improved the hatchability percentages of fertile eggs (*p* < 0.05) compared to the other injections. The improvement in hatchability may have been due to the high nutritive value of RJ, which contains vitamins and essential amino acids that enhance chick embryonic growth and hatchability. However, our results disagree with those obtained by Moghaddam et al. [21], who reported that in ovo, RJ injection significantly decreased hatchability (46.7%) compared to saline injection (68.3%). Moreover, Moghaddam et al. [22] found significantly lower hatchability percentages in eggs injected with RJ than in eggs injected with a saline phosphate antibiotic. Conclusively, RJ in ovo injections (0.5 mL RJ/egg) improved the hatchability percentages of chicken eggs in this study.

The results presented in Table 2 indicate that the El-Salam strain had significantly greater body weight and daily weight gain (*p* < 0.05) than the Dokki-4 strain, which may be attributable to its genetic makeup [28]. Injecting eggs with 0.5 mL RJ/egg significantly (*p* < 0.05) improved chicken body weight and daily weight gain compared to injecting eggs with saline. In agreement with our results, Ahangari et al. [23] reported that in ovo RJ injection elicited a significant positive effect on the body weight of broiler chicks at 21 days of age. RJ plays an important role in bee colonies, stimulating and increasing larval growth and metabolism [37] and some RJ bioactive components can affect crucial physiological processes [13,38]. Additionally, increased body weight has been observed after injection or ingestion of RJ in experimental animals [37]. The differences in body weight between the two strains at 8 weeks of age can be explained by the fact that at this age, this quantitative trait in chickens is affected by complex physiological mechanisms and multiple genetic factors [39]. Overall, RJ in ovo injections (0.5 mL RJ/egg) had limited beneficial effects on the body weights and weight gain of the chickens.

Consistent with the findings of Rondelli et al. [29], our results revealed improved feed consumption at 8–12 weeks of age (*p* < 0.05) and total feed consumption (0–12 weeks of age) (*p* < 0.0001) in the El-Salam strain compared to the Dokki-4 strain. No significant improvement in the feed conversion ratio (FCR) was recorded upon in ovo RJ injection. Ahangari et al. [23] found that in ovo RJ injection increased feed consumption and reduced the FCR. Seven et al. [40] reported that propolis and RJ enhanced growth performance measured as body weight, feed intake and FCR; these effects could be attributed to enhanced intestinal health, digestion and absorption due to the antimicrobial effects of the RJ and propolis components. The significant increases in dressing percentage upon treatment with 0.5 mL RJ/egg are consistent with results obtained by Moghaddam et al. [21], who found that in ovo RJ injection significantly increased dressed carcass percentages and heart and liver weights compared to a control treatment. There are several possible explanations for these results. First, RJ can increase oxygen metabolism and animal activity by increasing the concentration and use of blood glucose [41] and can also promote respiration and oxidative phosphorylation, increasing tissue oxygen consumption and, consequently, performance and endurance [42]. Furthermore, RJ also exhibits antioxidant properties [42] in addition to containing many dietary proteins with a wide range of functional and biological properties; some of these properties are attributable to biologically active peptides (of 2–20 amino acid residues) that are inactive when part of a protein but are activated when digested in vivo [43]. Feed consumption and giblet percentages were substantially affected by strain differences but dressing percentages were increased significantly in the group treated with 0.5 mL RJ/egg compared to the other groups.

Our results showed that there were no strain effects on the tested serum parameters. However, in ovo RJ injection decreased lipid profile parameters and increased total protein and globulin content compared to control saline injection (Table 3). A hypocholesterolaemic effect of RJ has also been reported by Kashima et al. [44], who suggested that the major identified RJ proteins (MRJP1, MRJP2 and MRJP3), as bile acid-binding proteins, significantly decreased the micellar solubility of cholesterol. Pavel et al. [45] confirmed the ability of RJ to reduce blood cholesterol and several studies have demonstrated the efficacy of RJ in lowering and controlling blood TGs and cholesterol levels [46,47]. Vittek [48] showed that administration of 50–100 mg RJ/d lowered serum total cholesterol levels by 14% and total lipids by 10%. A different study reported that ingestion of 6 g RJ/d for 4 weeks led to reduced serum total cholesterol and LDL but had no effect on HDL or TGs content [16,49]. The increased total protein, albumen and globulin in the RJ-treated groups may be attributable to a direct promoting impact of RJ on haemopoietic tissue in addition to a stimulatory anabolic effect on liver tissues that favours protein synthesis. In addition, RJ has been proven to protect against degeneration of body protein [50]. Our results are similar to those recorded by Mahmoud [51], who found that feeding of RJ to Ross broilers under different stocking densities increased serum total protein, albumen and globulin levels. Collectively, RJ in ovo injection (0.5 mL RJ/egg) had a hypocholesterolaemic effect on chickens in addition to a role in increasing total serum protein.

The results presented in Table 4 show that in ovo RJ injection increased the Hb percentage and lymphocyte count and decreased monocyte and eosinophil numbers compared to control saline injection. The increased lymphocyte count indicates that in ovo RJ injection improved the chicken response to stress. However, contradictory results were obtained by Ahangari et al. [23], who reported that RJ injection resulted in decreased lymphocyte counts and increased heterophil counts and heterophil/lymphocyte (HL) ratios. Additionally, Rabie et al. [52] reported that dietary or drinking water supplementation of Cobb 500 broiler chicks with propolis significantly increased Hb concentrations compared with a control treatment. However, Morita et al. [53] reported that there is a lack of articles that interpret the effect of RJ on anaemia. Recently, Bhalchandra et al. [54] reported that intraperitoneal injection of RJ in rats improved Hb concentrations and mean corpuscular haemoglobin (MCH) and suggested that honey and RJ exert protective effects against blood cell damage via preservation of cellular integrity. Our findings suggest that RJ feeding might have an anti-anaemic effect.

The results revealed a higher antibody titre against AIV (*p* < 0.05) and SRBCs (*p* < 0.0001) in the Dokki-4 strain than in the El-Salam strain (Table 5). The better immune response of Dokki-4 chickens to AIV and SRBCs may be attributable to the genetic potential inherited from the Fayoumi strain, a pure Egyptian native strain known globally for its strong immunity [28]. The effects of in ovo RJ injection on chicken immunity were unique (Table 5). RJ contains several forms of free amino acids at levels ranging from 0.6–1.5% and most are L-series amino acids, such as lysine and proline [55]. Administration of these amino acids influences the immune responses of poultry against disease, during which body proteins are broken down and the resultant amino acids are used for critical protein synthesis rather than growth, with consequent enhancement of defence against certain diseases [56]. Ahangari et al. [23] reported non-significant effects of RJ injection on day 14 of the experiment, although a significant increase in antibody titre against NDV was observed on day 28 of the trial. Generally, the Dokki-4 strain had a better immune response against AIV and SRBCs than the El-Salam strain; moreover, RJ in ovo injection (0.5 mL RJ/egg) improved the immunity of chickens against AIV, NDV and SRBCs.

Finally, with regard to the feasibility of RJ application in poultry production, RJ can be injected into eggs at trace concentrations (not exceeding 0.5 mg/egg). Poultry production depends on both input and output; the marked improvements in bird growth characteristics in the present study as a result of RJ treatment suggest that even if the price of RJ is high, the economic return will cover the cost of its use and even yield a good profit margin for chicken keepers.

## 5. Conclusions

On the basis of our results, the study hypothesis was accepted that varying the chicken strain could alter the response to the in ovo injection with RJ (the Dokki-4 strain was superior to the El-Salam strain for the tested parameters). RJ injection into the yolk sac elicited significant positive effects on hatching parameters, growth performance, blood chemistry, haematology and immunological parameters. Among the injected doses, the 0.5 mL dose of RJ resulted in the best hatching parameters, growth performance and immune and health-related traits.

## Figures and Tables

**Table 1:** Fertility and hatchability percentages of two chicken strains (El-Salam and Dokki-4) injected in ovo with two different levels of royal jelly (RJ; 0.25, 0.5 mL/egg) compared to counterpart control strains (0 mL RJ/egg) and the interaction between strain and treatment level.

Items		Number of Replicates	Fertility (%)	Hatchability (%) (Based on Fertile Eggs)
Strain Effect				
El-Salam		18	84.09 ^b^	81.09
Dokki-4		18	85.64 ^a^	83.81
RJ in ovo Injection Effect (mL/egg)				
0		12	84.83	79.75 ^b^
0.25		12	84.95	82.28 ^ab^
0.50		12	84.82	85.32 ^a^
Interaction Effect				
Strain	RJ in ovo Injection			
El-Salam	0	6	84.07	78.93
	0.25	6	84.2	81.13
	0.5	6	84	83.2
Dokki-4	0	6	85.6	80.57
	0.25	6	85.7	83.43
	0.5	6	85.63	87.43
SEM			0.76	1.86
*p*-Value				
Strain		-	0.028	0.097
In ovo injection		-	0.982	0.034
Strain × in ovo injection		-	0.995	0.771

SEM—standard error of the mean. In the same column and within the same effect, means with different superscripts (^a, b^) differ significantly (*p* < 0.05).

**Table 2:** Effect of in ovo injection of royal jelly (RJ) at two levels (0.25, 0.5 mL/egg) on growth performance and carcass parameters of two chicken strains (El-Salam, Dokki-4) as compared to counterpart control chicks (0 mL/egg) and the interaction of strain and treatment levels.

Items		Number of Replicates	Daily Weight Gain (DWG, g/bird)	Feed Consumption (FC, g)	Feed Conversion Ratio (FCR, g Feed/g Gain)	Carcass Parameters (% of Live Weight)	
			0–12 Weeks	0–12 Weeks	0–12 Weeks	Dressing	Giblets
Strain Effect							
El-Salam		18	35.38	56.53 ^a^	1.62	71.50	5.53 ^b^
Dokki-4		18	31.76	51.89 ^b^	1.67	70.17	7.37 ^a^
RJ in ovo Injection Effect (mL/egg)							
0		12	32.22	53.20	1.67	68.37 ^b^	7.13
0.25		12	33.52	54.53	1.67	71.62 ^ab^	6.93
0.50		12	34.98	54.90	1.60	72.52 ^a^	5.93
Interaction Effect							
Strain	RJ in ovo Injection						
El-Salam	0	6	33.67	55.03	1.67	69.43	5.97
	0.25	6	35.6	57.3	1.63	71.8	5.37
	0.5	6	36.87	57.27	1.57	73.27	5.27
Dokki-4	0	6	30.6	51.37	1.67	67.3	8.3
	0.25	6	31.43	51.77	1.67	71.43	7.23
	0.5	6	32.83	52.53	1.6	71.77	6.6
SEM			2.12	0.68	0.12	1.01	0.46
*p*-value						
Strain			0.058	0.0001	0.65	0.273	0.004
In ovo injection			0.449	0.063	0.808	0.031	0.211
Strain × in ovo injection			0.954	0.411	0.947	0.823	0.751

SEM—standard error of the mean. In the same column and within the same effect, means with different superscripts (^a, b^) differ significantly (*p* < 0.05).

**Table 3:** Effect of in *ovo* injection of royal jelly (RJ) at two levels (0.25 and 0.5 mL/egg) on the serum lipid and protein profiles of two chicken strains (El-Salam and Dokki-4) compared to control saline injection (0 mL RJ/egg) and the interaction between strain and treatment level.

Parameter		Number of Samples	Total Lipids (mg/dL)	Cholesterol (mg/dL)	TGs (mg/dL)	HDL (mg/dL)	LDL (mg/dL)	TP (g/dL)	Albumen (g/dL)	Globulin (g/dL)	AG Ratio
Strain Effect											
El-Salam		30	231.89	114.32	103.64	44.52	80.19	4.63	1.93	2.69	0.72
Dokki-4		30	227.50	111.72	103.87	43.80	78.93	4.80	2.03	2.77	0.73
RJ in ovo injection effect (mL/egg)											
0		20	236.15 ^a^	125.60 ^a^	106.13 ^a^	46.90 ^a^	85.05 ^a^	4.22 ^b^	1.81	2.41 ^c^	0.75
0.25		20	228.50 ^a^	108.38 ^b^	103.18 ^b^	43.50 ^b^	77.65 ^b^	4.71 ^ab^	1.99	2.72 ^b^	0.73
0.5		20	224.43 ^b^	105.08 ^b^	101.95 ^b^	42.08 ^b^	75.98 ^b^	5.21 ^a^	2.14	3.07 ^a^	0.70
Interaction Effect											
Strain	RJ in ovo Injection										
El-Salam	0	10	240.6	128.33	106.67	47.6	85.7	4.13	1.78	2.35	0.75
	0.25	10	229.77	109.27	102.77	43.8	78.5	4.77	1.98	2.79	0.71
	0.5	10	225.3	105.37	101.5	42.17	76.37	4.98	2.05	2.94	0.7
Dokki-4	0	10	231.7	122.87	105.6	46.2	84.4	4.32	1.86	2.46	0.75
	0.25	10	227.23	107.5	103.6	43.2	76.8	4.65	2	2.65	0.75
	0.5	10	223.57	104.8	102.4	42	75.6	5.44	2.23	3.21	0.69
SEM			3.39	2.36	3.39	2.36	1.18	1.5	3.31	0.28	0.21
*p*-Value											
Strain			0.139	0.201	0.82	0.567	0.65	0.468	0.601	0.4376	0.847
In ovo injection			0.014	0.0001	0.011	0.021	0.04	0.014	0.354	0.0005	0.72
Strain × In ovo injection			0.53	0.57	0.647	0.917	0.99	0.604	0.93	0.262	0.936

TGs—triglycerides; HDL—high-density lipoproteins; LDL—low-density lipoproteins; TP—total protein; AG—albumin/globulin; SEM—standard error of the mean. In the same column and within the same effect, means with different superscripts (^a, b, c^) differ significantly (*p* < 0.05).

**Table 4:** Effect of in ovo injection of royal jelly (RJ) at two levels (0.25, 0.5 mL/egg) on complete blood count (CBC) of two chicken strains (El-Salam, Dokki-4) as compared to counterpart control chicks (0 mL/egg) and the interaction of strain and treatment levels.

Parameter		Number of Samples	RBCs (10^6^/mm^3^)	PCV (%)	Hb (g %)	WBCs (10^3^/mm^3^)	Heterophils (%)	Lymphocytes (%)	HL Ratio	Monocytes (%)	Eosinophils (%)	Basophils (%)
Strain Effect												
El-Salam		30	2.17	32.24	18.32	4.31	21.89	71.50	46.70	4.80	1.70	3.03
Dokki-4		30	2.19	32.08	18.61	4.42	21.82	71.91	46.87	4.72	1.63	2.80
RJ in ovo Injection Effect (mL/egg)												
0		20	2.05	31.50	16.49 ^b^	4.27	22.39	68.81 ^b^	45.60	6.42 ^a^	1.91	3.30 ^a^
0.25		20	2.23	32.33	18.93 ^a^	4.28	21.76	72.56 ^a^	47.16	4.61 ^b^	1.59	2.88 ^ab^
0.5		20	2.25	32.64	19.97 ^a^	4.56	21.42	72.74 ^a^	47.58	3.26 ^c^	1.52	2.57 ^b^
Interaction Effect												
Strain	RJ in ovo Injection											
El-Salam	0	10	2.04	31.61	16.31	4.16	22.46	68.84	45.65	6.27	1.97	3.5
	0.25	10	2.22	32.31	18.72	4.25	21.78	72.27	47.03	4.79	1.56	3.1
	0.5	10	2.25	32.8	19.92	4.53	21.43	73.39	47.41	3.33	1.57	2.5
Dokki-4	0	10	2.05	31.4	16.68	4.37	22.32	68.78	45.55	6.56	1.83	3.1
	0.25	10	2.24	32.35	19.13	4.31	21.74	72.85	47.29	4.42	1.61	2.65
	0.5	10	2.25	32.48	20.02	4.58	21.41	74.1	47.75	3.18	1.46	2.63
SEM			0.12	0.12	1.17	0.65	0.17	0.69	1.07	0.87	0.42	0.21
*p*-Value												
Strain			0.936	0.868	0.594	0.439	0.902	0.648	0.815	0.832	0.703	0.221
In ovo injection			0.194	0.613	0.0005	0.183	0.386	0.001	0.093	0.0001	0.196	0.022
Strain × in ovo injection			0.993	0.967	0.967	0.867	0.996	0.929	0.963	0.737	0.891	0.389

RBCs—red blood cells, PCV—packed cell volume, Hb—haemoglobin, WBCs—white blood cells, HL—heterophil/lymphocyte, SEM—standard error of the mean. In the same column and within the same effect, means with different superscripts (^a, b, c^) differ significantly (*p* < 0.05).

**Table 5:** Effect of in ovo injection of royal jelly (RJ) at two levels (0.25, 0.5 mL/egg) on Antibody titre against AIV, NDV and SRBCs of two chicken strains (El-Salam, Dokki-4) as compared to counterpart control (0 mL/egg) and the interaction of strain and treatment levels.

Parameter		Number of Samples	AIV 12 Weeks	NDV		SRBCs
				7 Weeks	12 Weeks	
Strain Effect						
El-Salam		30	5.77 ^b^	6.50	6.70	6.18 ^b^
Dokki-4		30	6.22 ^a^	6.72	7.04	7.41 ^a^
RJ in ovo Injection Effect (mL/egg)						
0		20	4.55 ^c^	6.10 ^b^	6.25 ^b^	6.33 ^b^
0.25		20	6.41 ^b^	6.20 ^b^	6.57 ^b^	6.33 ^b^
0.5		20	7.03 ^a^	7.53 ^a^	7.80a	7.74 ^a^
Interaction Effect						
Strain	RJ in ovo Injection					
El-Salam	0	10	4.5	6	6.2	4.95 ^d^
	0.25	10	5.99	6.1	6.3	6.23 ^c^
	0.5	10	6.82	7.4	7.6	7.37 ^b^
Dokki-4	0	10	4.6	6.2	6.3	6.43 ^c^
	0.25	10	6.82	6.3	6.83	7.70 ^ab^
	0.5	10	7.23	7.67	8	8.11 ^a^
SEM			0.22	0.15	0.22	0.15
*p*-Value						
Strain			0.027	0.097	0.051	0.0001
In ovo injection			0.0001	0.0001	0.0001	0.0001
Strain × in ovo injection			0.283	0.968	0.538	0.0001

AIV—avian influenza virus; NDV—Newcastle disease virus; SRBCs—sheep red blood cells; SEM—standard error of the mean. In the same column and within the same effect, means with differ superscripts (^a, b, c, d^) differ significantly (*p* < 0.05).

## References

[1] Uni Z., Ferket P.R., Tako E., Kedar O. (2005). *In ovo* feeding improves energy status of late-term chicken embryos. Poult. Sci..

[2] Foye O.T., Uni Z., Ferket P.R. (2006). Effect of *in ovo* feeding egg white protein, beta-hydroxy-beta-methylbutyrate and carbohydrates on glycogen status and neonatal growth of turkeys. Poult. Sci..

[3] Kadam M., Bhanja S., Mandal A., Thakur R., Vasan P., Bhattacharyya A., Tyagi J., Bhanja S. (2008). Effect ofin ovothreonine supplementation on early growth, immunological responses and digestive enzyme activities in broiler chickens. Br. Poult. Sci..

[4] Zhai W., Neuman S., Latour M.A., Hester P.Y. (2008). The Effect of *in ovo* Injection of L-Carnitine on Hatchability of White Leghorns. Poult. Sci..

[5] Keralapurath M.M., Corzo A., Pulikanti R., Zhai W., Peebles E.D. (2010). Effects of *in ovo* injection of L-carnitine on hatchability and subsequent broiler performance and slaughter yield. Poult. Sci..

[6] Ebrahimnezhad Y., Salmanzadeh M., Habib A., Rahim B., Rahimi H. (2011). The effects of *in ovo* injection of glucose on characters of hatching and parameters of blood in broiler chickens. Ann. Biol. Res..

[7] Saeed M., Babazadeh D., Naveed M., Alagawany M., El-Hack M.E.A., Arain M.A., Tiwari R., Sachan S., Karthik K., Dhama K. (2019). *In ovo* delivery of various biological supplements, vaccines and drugs in poultry: Current knowledge. J. Sci. Food Agric..

[8] Bhanja S.K., Mandal A.B., Goswami T.K. (2004). Effect of *in ovo* injection of amino acids on growth, immune response, development of digestive organs and carcass yields of broilers. Indian J. Poult. Sci..

[9] Bhanja S.K., Mandal A.B. (2005). Effect of *In ovo* Injection of Critical Amino Acids on Pre- and Post-hatch Growth, Immunocompetence and Development of Digestive Organs in Broiler Chickens. Asian Australas. J. Anim. Sci..

[10] Simuth J., Bilikova K., Kovacora E. Royal Jelly proteins as a tool for development of functional ingredients for health. Proceedings of the 38th Congress of Apimondia.

[11] Ramadan M.F., Al-Ghamdi A., Hassanien P.M.F.R. (2012). Bioactive compounds and health-promoting properties of royal jelly: A review. J. Funct. Foods.

[12] Stöcker A., Schramel P., Kettrup A., Bengsch E. (2005). Trace and mineral elements in royal jelly and homeostatic effects. J. Trace Elem. Med. Biol..

[13] Fujiwara S., Imai J., Fujiwara M., Yaeshima T., Kawashima T., Kobayashi K. (1990). A potent antibacterial protein in royal jelly. Purification and determination of the primary structure of royalisin. J. Biol. Chem..

[14] Wei W., Wei M., Kang X., Deng H., Lu Z. (2009). A novel method developed for acetylcholine detection in royal jelly by using capillary electrophoresis coupled with electrogenerated chemiluminescence based on a simple reaction. Electrophoresis.

[15] Nakajima Y., Tsuruma K., Shimazawa M., Mishima S., Hara H. (2009). Comparison of bee products based on assays of antioxidant capacities. BMC Complement. Altern. Med..

[16] Guo H., Saiga A., Sato M., Miyazawa I., Shibata M., Takahata Y., Morimatsu F. (2007). Royal Jelly Supplementation Improves Lipoprotein Metabolism in Humans. J. Nutr. Sci. Vitaminol..

[17] Kohno K., Okamoto I., Sano O., Arai N., Iwaki K., Ikeda M., Kurimoto M. (2004). Royal Jelly Inhibits the Production of Proinflammatory Cytokines by Activated Macrophages. Biosci. Biotechnol. Biochem..

[18] Townsend G.F., Morgan J.F., Tolnai S., Hazlett B., Morton H.J., Shuel R.W. (1960). Studies on the in vitro antitumor activity of fatty acids. I. 10-Hydroxy-2-decenoic acid from royal jelly. Cancer Res..

[19] Tseng J.M., Huang J.R., Huang H.C., Tzen J.T., Chou W.M., Peng C.C. (2011). Facilitative production of an antimicrobial peptide royalisin and its antibody via an artificial oil-body system. Biotechnol. Prog..

[20] Park H.M., Cho M.H., Cho Y., Kim S.Y. (2012). Royal Jelly Increases Collagen Production in Rat Skin after Ovariectomy. J. Med. Food.

[21] Moghaddam A.A., Borji M., Komazani D. (2014). Hatchability rate and embryonic growth of broiler chicks following *in ovo* injection royal jelly. Br. Poult. Sci..

[22] Moghaddam A., Karimi I., Borji M., Bahadori S., Abdolmohammadi A. (2013). Effect of royal jelly *in ovo* injection on embryonic growth, hatchability, and gonadotropin levels of pullet breeder chicks. Theriogenology.

[23] Ahangari Y.J., Hashemi S.R., Akhlaghi A., Atashi H., Esmaili Z., Ghorbani M., Mastani R., Azadegan A., Davoodi H. (2013). Effect of *in ovo* Injection of Royal Jelly on Post-Hatch Growth Performance and Immune Response in Broiler Chickens Challenged with Newcastle Disease Virus. Iran. J. Appl. Anim. Sci..

[24] El-Tarabany M.S. (2018). Effect of Royal Jelly on behavioural patterns, feather quality, egg quality and some haematological parameters in laying hens at the late stage of production. J. Anim. Physiol. Anim. Nutr..

[25] Deeb N., Lamont S.J. (2002). Genetic Architecture of growth and body weight composition in unique chicken population. J. Hered..

[26] Ajayi F.O., Agaviezor B.O. (2016). Fertility and Hatchability Performance of Pure and Crossbred Indigenous Chicken Strains in the High Rainforest Zone of Nigeria. Int. J. Livest. Prod..

[27] Shim M.Y., Karnuah A.B., Tahir M., Miller M., Pringle T.D., Aggrey S.E., Pesti G.M. (2012). Strain and sex effects on growth performance and carcass traits of contemporary commercial broiler crosses. Poult. Sci..

[28] Taha A.E., El-Edel M.A., El-Lakany H.F., Shewita R.S. (2012). Growth Performance and Immune Response against Newcastle and Avian Influenza Vaccines in Egyptian Chicken Strains. Glob. Vet..

[29] Rondelli S., Martinez O., García P. (2003). Sex effect on productive parameters, carcass and body fat composition of two commercial broilers lines. Rev. Bras. Cienc. Avic..

[30] Ojedapo L., Akinokun O., Adedeji T., Olayeni T., Ameen S., Ige A., Amao S. (2008). Evaluation of Growth Traits and Short-Term Laying Performance of Three Different Strains of Chicken in the Derived Savannah Zone of Nigeria. Int. J. Poult. Sci..

[31] Zhao J.P., Chen J.L., Zhao G.P., Zheng M.Q., Jiang R.R., Wen J. (2009). Live performance, carcass composition, and blood metabolite responses to dietary nutrient density in two distinct broiler breeds of male chickens. Poult. Sci..

[32] El-Itriby A.A., Sayed I.F. (1996). “Dokki-4” A new strain of poultry. Agric. Res. Rev..

[33] Abd El-Gawad E.M., Balat M.M., Abo El-Ela N.Y., Ali M.M., Omran K.M. (1983). “El-Salam” A new locally developed strain of chickens. Agric. Res. Rev..

[34] Wegmann T.G., Smithies O. (1996). A simple hemagglutination system requiring small amounts of red cells and antibodies. Transfusion.

[35] Akiba Y., Jensen L.S., Bart C.R., Kraeling R.R. (1982). Plasma estradiol, thyroid hormones and liver lipids determination in birds. J. Nut..

[36] SAS (2004). Statistical User’s Guide, INT..

[37] Bonomi A., Bonomi B.M., Quarantelli A. (2001). Royal jelly in turkey feeding; Royal jelly in guinea-fowl feeding. Riv. Sci. Anim..

[38] Wu G., Bíliková K., Šimúth J. (2001). Isolation of a peptide fraction from honeybee royal jelly as a potential antifoulbrood factor. Apidologie.

[39] Siegel P.B. (1962). Selection for Body Weight at Eight Weeks of Age: 1. Short term Response and Heritabilities. Poult. Sci..

[40] Seven P.T., Arslan A.S., Özçelik M., Şimşek Ü.G., Seven İ. (2016). Effects of propolis and royal jelly dietary supplementation on performance, egg characteristics, lipid peroxidation, antioxidant enzyme activity and mineral levels in Japanese quail. Europ. Poult. Sci..

[41] Gonnard P., N’guyen C.C. (1957). Action of royal jelly on oxygen consumption in tissues in vitro. Ann. Pharm. Françaises.

[42] Krylov V., Sokolskii C. (2000). Royal jelly. Agroprompoligraf. Krasn..

[43] Cheison S.C., Wang Z. (2003). Bioactive milk peptides: Rede-fining the food-drug interphase. A review. Afr. J. Food Nutr. Sci. Agric. Dev..

[44] Kashima Y., Kanematsu S., Asai S., Kusada M., Watanabe S., Kawashima T., Nakamura T., Shimada M., Goto T., Nagaoka S. (2014). Identification of a Novel Hypocholesterolemic Protein, Major Royal Jelly Protein 1, Derived from Royal Jelly. PLoS ONE.

[45] Pavel C.I., Mărghitaş L.A., Bobiş O., Dezmirean D.S., Şapcaliu A., Radoi I., Mădaş M.N. (2011). Biological activities of royal jelly-review. Sci. Pap. Anim. Sci. Biotech..

[46] Cho Y.T. (1977). Studies on royal jelly and abnormal cholesterol and triglycerides. Am. Bee J..

[47] Pizzorno J., Murray M., Joiner-Bey H. (2007). The Clinician’s Handbook of Natural Medicine.

[48] Vittek J. (1995). Effect of Royal Jelly on serum lipids in experimental animals and humans with atherosclerosis. Cell. Mol. Life Sci..

[49] Kamakura M., Suenobu N., Fukushima M. (2001). Fifty-seven-kDa Protein in Royal Jelly Enhances Proliferation of Primary Cultured Rat Hepatocytes and Increases Albumin Production in the Absence of Serum. Biochem. Biophys. Res. Commun..

[50] Elnagar S.A., Elghalid O.A., Abd-Elhady A.M. (2010). Royal jelly: Can it reduce physiological strain of growing rabbits under Egyptian summer conditions?. Animal.

[51] Mahmoud F.A. (2016). Impact of Chinese Royal Jelly on Performance, Behaviour and Some Blood Parameters in Broilers Reared under High Stocking Density. Asian J. Anim. Vet. Adv..

[52] Rabie A.H., El-Kaiaty A.M., Hassan M.S.H., Stino F.K.R. (2018). Influence of some honey bee products on some haematological and immunological parameters and meat quality in broilers. Egypt. Poult. Sci..

[53] Morita H., Ikeda T., Kajita K., Fujioka K., Mori I., Okada H., Uno Y., Ishizuka T. (2012). Effect of royal jelly ingestion for six months on healthy volunteers. Nutr. J..

[54] Bhalchandra W., Alqadhi Y.A., Ninawe A.S. (2018). Ameliorative Role of Bee Honey and Royal Jelly against Cisplatin Induced Alteration in Hematological Parameters in Male Wister Albino Rat. Int. J. Pharm. Pharm. Sci..

[55] Boselli E., Caboni M.F., Sabatini A.G., Marcazzan G.L., Lercker G. (2003). Determination and changes of free amino acids in royal jelly during storage. Apidologie.

[56] Hashemi S., Maroufyan E., Kasim A., Loh T., Bejo M. (2010). Responses of Performance and Differential Leukocyte Count to Methionine and Threonine Supplementations on Broiler Chickens Challenged with Infectious Bursal Disease in Tropical Condition. Asian J. Biol. Sci..

